# Effect of self-employment on the sub-health status and chronic disease of rural migrants in China

**DOI:** 10.1186/s12889-021-12214-5

**Published:** 2021-12-11

**Authors:** Jian Zhou, Qiushi Wu, Zicheng Wang

**Affiliations:** School of Public Administration, Jinan University, 601 Huangpu Blvd, Tianhe District, Guangzhou, 510632 Guangdong Province China

**Keywords:** Self-employment, Sub-health status, Chronic disease, Rural migrants, Social health insurance program

## Abstract

**Background:**

Rural migrants usually suffer from major disease risks, but little attention had been paid toward the relationship between self-employment behavior and health status of rural migrants in China. Present study aims to explore the causal effect of self-employment behavior on rural migrants’ sub-health status and chronic disease. Two research questions are addressed: does self-employment status affect the sub-health status and chronic disease of rural migrants? What is potential mechanism that links self-employment behavior and health status among rural migrants in China?

**Methods:**

The dataset from the 2017 National Migrants Population Dynamic Monitoring Survey (NMPDMS-2017) was used to explore the causal effect. Logit regression was performed for the baseline estimation, and linear probability model with instrument variable estimation (IV-LPM) was applied to correct the endogeneity of self-employment. Additionally, logit regression was conducted to explore the transmission channel.

**Results:**

Self-employed migrants were more susceptible to sub-health status and chronic disease, even when correcting for endogeneity. Moreover, self-employed migrants were less likely to enroll in social health insurance than their wage-employed counterparts in urban destinations.

**Conclusion:**

Self-employed migrants were more likely to suffer from sub-health status and chronic disease; thus, their self-employment behavior exerted a harmful effect on rural migrants’ health. Social health insurance may serve as a transmission channel linking self-employment and rural migrants’ health status. That is, self-employed migrants were less prone to participate in an urban health insurance program, a situation which leaded to insufficient health service to maintain health.

## Background

Since 1978, China has experienced rapid and unprecedented urbanization in which millions of migrants move from rural to urban areas. Rural migrants have become an important segment of industrial workers and have made great contributions to urban development [1, 2]. Nevertheless, rural migrants often work in the secondary urban labor market, which involves lower income, unstable jobs, longer work hours, and the lack of occupational health protection [3, 4]. Such situation not only affects the socio-economic status of rural migrants in urban destinations, but may also produce negative effects on rural migrants’ health [4, 5]. Previous studies have revealed that rural migrants usually suffer from major disease risks, especially occupational diseases, chronic illnesses, and sexually transmitted diseases [6, 7].

To improve their socio-economic status, a large proportion of rural migrants choose to engage in self-employment [8], which is defined as proprietors of privately or individually-owned businesses with no hired labor following the Social Insurance Law of the People’s Republic of China in 2011. Self-employment is often characterized by high work autonomy, flexibility and skill utilization as well as high income [9, 10]. Nevertheless, self-employment often involves considerable uncertainty in business activities (such as investment risk, market fluctuation, and irregular working hours), which may cause unhealthy behaviors [11]. Thus, a rich array of literature has explored the effect of self-employment on health, but no consensus has been reached in empirical studies. The job demand–control model posits that job demand increases the work-related stress of the self-employed, whereas their higher job control reduces it. That is, job control can weaken the negative relationship between self-employment and work-related stress; thus, the self-employed are healthier than their wage-employed counterparts [12]. In addition, the independence, autonomy, and high compensation from self-employment may induce life satisfaction [13, 14]. A growing body of evidence confirmed that self-employment status had a positive impact on health status [12, 15, 16]. Furthermore, self-employed individuals experienced better health than wage-employed employees [17–19].

A few studies also revealed the contrary conclusion that self-employment was negatively related with health status [20, 21]. Self-employed people were often confronted with unanticipated demand shocks, a circumstance that subjected them to high workload and volatile earning flows, which, in turn, had been implicated as causes of stress. Work-related stress not only deteriorated performance at work but may also impair the health status of the self-employed [22, 23]. Work-related stress was also associated with unhealthy behaviors such as smoking and drinking, and these habits may further deteriorate workers’ health status [24, 25]. In addition, self-employment only exerted a positive effect on perceived health, but had a negative effect on workers’ objective health status [26].

The potential causes of those inconsistent conclusions may be due to endogeneity, which posited that self-employment and health were mutually influenced. Giandrea, Cahill and Quinn [27] found that poor health status had negatively impact on individual self-employment decision. However, the existing studies did not address this endogeneity [12, 15], and the instrumental variable approach should be applied to present an unbiased estimation. Furthermore, the mechanism linking self-employment status and health was underexplored.

Previous research in China mainly focused on the determinants of self-employment decision, such as the institutional environment and social networks [28, 29]. Little attention had been paid toward the relationship between self-employment behavior and health status of rural migrants, and discussion on the transmission channel linking self-employment and rural migrants’ health remains scant.

However, China currently faces serious health challenges. Sub-health status has become a new public health issue in China. It refers to an intermediate health state between health and disease, and it is characterized by a decline in vitality, physiological function, and capacity for adaption; this status is regarded as a subclinical, reversible stage of chronic disease [30, 31]. The number of people who reported suboptimal health status, that is, poor health in the absence of a diagnosable condition, has increased in China in recent years. Thus, studies on improving the intervention and prognosis for sub-health status have become increasingly important. According to the blue book of health management, chronic diseases numbered approximately 300 million in 2018 [32]. In the “Thirteenth Five-Year Plan,” chronic disease health management has been upgraded to a national strategic height. Thus, chronic diseases have become an important public health issue and have attracted increasing attention from scholars and policy makers. Hypertension and diabetes were two of the most common chronic diseases according to Tilov, Semerdzhieva, Bakova, Tornyova and Stoyanov [33] and DeVol et al. [29]. Healthy China Initiative (2019–2030) showed that 270 million people had hypertension and more than 97 million had diabetes in China. Therefore, hypertension and diabetes were chosen as proxies for chronic disease in this study. As a unique group, little attention has been given to the health status (including chronic diseases and sub-health status) of self-employed rural migrants in China. Therefore, this study focused on the effect of self-employment on the health status of rural migrants, including two sets of health indicators, namely, sub-health status and chronic disease.

This study addressed these gaps by exploring two issues: does self-employment status affect the sub-health status and chronic disease of rural migrants? If so, what is the potential mechanism that links self-employment behavior and health status?

This research contributed to the literature in three distinct ways. First, this study was unique, given its focus on internal migrant groups. A comprehensive database in China was used to explore the direct association between self-employment behavior and sub-health status as well as the chronic disease of rural migrants. Second, this research applied linear probability model with instrument variable to correct the potential endogeneity of self-employment in order to identify the precise causal effects of self-employment behavior on health status. By comparison, previous studies merely explored the correlation by using multiple non-linear regression analysis. Third, the potential transmission channels linking self-employment status and health were discussed in the context of China by estimating the effect of self-employment on social health insurance.

## Methods

### Study design

Data from the 2017 National Migrants Population Dynamic Monitoring Survey (NMPDMS-2017) was analyzed in this work. In this survey, the stratified multistage random sampling method with the probability proportional to size approach was employed to extract sampling points from 31 provinces and the Xinjiang Production and Construction Corps (XPCC) in China. These samples included internal migrants aged 15–69 years who did not have the local “household registration system (Hukou),” an institution with the power to restrict population mobility and access to local public benefit for rural population, and have been living in destination cities for more than 1 month. The NMPDMS-2017 survey had two features that made it particularly suitable for our research. First, the sample size was large, which contained 169,989 rural migrants. Second, it collected a wide variety of data related to the demography, employment traits, and health status among rural migrants.

As this study aimed to investigate the effect of self-employment on the health status of rural migrants including the self-employed and wage workers that had rural household registrations, participants who were employers, temporary workers, or unemployed and those without rural household registrations were excluded. According to the definition of migrants and after dropping missing data, we obtained 114,675 valid samples, including 39,937 self-employed and 74,738 wage-employed rural migrants.

### Self-employment assessment

The definition of self-employment varied slightly across countries. A rich array of studies was conducted on the basis of official data sets for which the definition of self-employment is similar to that adopted by the International Labour Organization (ILO) [34]. According to the ILO, self-employment comprised three specific groups: self-employed workers with employees (employers), self-employed workers without employees (own-account workers), and members of producers’ cooperatives and contributing family workers. Following ILO, the Social Insurance Law of the People’s Republic of China in 2011 regarded self-employment as a part of flexible employment and defined the self-employed as proprietors of privately or individually owned businesses with no hired labor.

Thus, rural migrants who ran privately or individually owned businesses with no hired labor were identified as the self-employed in this work. The item for employment status was used to define self-employment, such that participants who ran their own businesses without employees were coded as 1, and as 0 if otherwise.

### Health measure

Two sets of health indicators, sub-health status and chronic disease, were applied to measure the health status of rural migrants.

Sub-health status in the study was assessed through the question “How is your health?” Responses were coded as 1 if the participant reported his/her health status between health and illness, and as 0 if otherwise. In addition, the definition of chronic disease was derived from the item “Have you been diagnosed with hypertension or diabetes?” Participants who suffered from hypertension or diabetes were coded as 1, and as 0 if otherwise.

### Potential covariates

In line with Rietveld, Kippersluis, and Thurik [35] and Wong et al. [36], the potential covariates in this work were categorized as socioeconomic characteristics (i.e., gender, age, age-squared, education attainment, income, and marital status), work characteristics, and migration traits (i.e., those who migrated with their children and those who migrated with their spouse). Descriptions of the measures were presented in Table 1.

**Table 1:** Operationalization of variables

Variable	Description	Items
**Dependent variables**		
Chronic disease	=1 if one suffer from Hypertension or Diabetes; =0 otherwise;	Question: Do you suffer from Hypertension or Diabetes diagnosed by your doctor?
Sub-health status	=1 if one is in sub-health status; =0 otherwise;	Question: How is your health?
**Focal variable**		
Self-employment	=1 if one is self-employed; =0 if wage-employed;	Question: What is your employment status?
**Control variables**		
Socio-economic characteristics		
Gender	=1 if male; =0 if female;	Question: What is your gender?
Age	15 ≤ age ≤ 96;	Question: Year of birth
Age^2	Age squared;	Question: Year of birth
Educational attainment		
Primary school or below	=1 if education year≤6, =0 otherwise	Question: What’s your education level?
Junior high school	=1 if 6 < education year≤9, =0 otherwise	Question: What’s your education level?
Senior high school	=1 if 9 < education year≤12, =0 otherwise	Question: What’s your education level?
College or above	=1 if education year> 12, =0 otherwise	Question: What’s your education level?
Income (monthly), Ұ	Net income for last month at time of interview	Question: How much was your salary last month?
Marital status	=1 if married;=0 if unmarried;	Question: What’s your marital status?
Work characteristic		
Construction industry	=1 if one works in construction industry;=0 otherwise;	Question: What’s do you work in industry?
Service industry	=1 if one works in service industry;=0 otherwise;	Question: What’s do you work in industry?
Other industry	=1 if one works in other industry;=0 works in construction or service industry;	Question: What’s do you work in industry?
Migration traits		
Children migration	=1 if children migrates together only; =0 otherwise	Question: What is your relationship with the interviewee?
Couple migration	=1 if couple migrates together only; =0 otherwise	Question: What is your relationship with the interviewee?

### Instrumental variables

As discussed before, the health status of rural migrants might affect their self-employment decision [37], that is, those with poor health face more difficulty in being self-employed, which have to bear higher levels of stress and working hours [38]. In order to address the bias resulting from this simultaneity, the study employed linear probability model with instrument variable estimation as our empirical approach.

We aggregated individual-level self-employment at the provincial level with sample weights to construct provincial self-employment rate as our instrument variable. We would define the measure of the provincial self-employment rate and discuss the rationale for this choice in next section.

### Model strategy

Since our main dependent variable was binary health indicators, the results of logit model were more accurate comparing with linear probability model, a binary logit model was applied to explore the effect of self-employment behavior on rural migrants’ health status. The following reduced form equation serves as the benchmark model:

1 $${H}_i=\alpha +\phi {self}_i+\beta {x}_i+{\varepsilon}_i,$$

Where *H*_*i*_ measured two sets of health indicators, namely chronic disease (equal to 1 if migrants suffered from hypertension or diabetes, and 0 if otherwise) and sub-health status (equal to 1 if migrants suffered from sub-health status, and 0 if otherwise). Focal variable *self*_*i*_ was a dummy variable representing whether the rural migrants were self-employed or not. *x*_*i*_ controlled for various socio-demographic characteristics, work characteristics and migration-related traits that may affect health status. Finally, *ε*_*i*_ captured the random error.

The effect of self-employment on health status may be biased because of the reverse causality in the logit estimation, and  self-employment in Eq. (1) was a dummy variable, thus IV-LPM may be appropriate in the study.^1^

2 $${self}_i=\kern0.5em {\delta}_0+{\delta}_1{x}_i^{\prime }+{\delta}_2{\mathrm{Z}}_i^{\prime }+{\nu}_i,$$

Where *self*_*i*_ was a dummy variable for self-employed status, ${x}_i^{\prime }$ incorporated the control variables, *ν*_*i*_ was the random error, and ${\mathrm{Z}}_i^{\prime }$ represented the instrument variable.

A valid instrument of self-employment should meet two criteria: it must be strongly related with the self-employed and cannot be associated with *ν*_*i*_. We chose provincial self-employment rate as the equivalent instrument, which was calculated from the NMPDMS-2017 survey. Since the NMPDMS-2017 survey obtained samples using a stratified multistage random sampling method with the probability proportional to size approach, we applied individual standardized weights (*ω*_*i*_) to each sample to improve the accuracy of the estimation when calculating provincial self-employment rate. The measurement of provincial self-employment rate was as followed. Firstly, we calculated the sum of self-employed individuals in a province by weighting, *S*_*j*_ =  ∑ *self*_*i*_*ω*_*i*_ (*self* = 0, 1, *j* = 1, …, 32); Secondly, we calculated the sum of total samples in a province by weighting, *P*_*j*_ =  ∑ *Iω*_*i*_ (*I* = 1); Finally, the provincial self-employment rate equaled the sum of the weight of the number of the self-employed (*S*_*j*_) dividing by the weight of total samples in a province (*P*_*j*_), $Z_j=\frac{S_j}{P_j}$.

### The choice of the instrumental variables

The potential reasons for instrument selection were as follows. Provincial self-employment rate represented the vitality of innovation and entrepreneurship in urban destinations and had a direct impact on individuals’ self-employment behaviors. Most of the regional policies in China focused on supporting entrepreneurs, which directly affected individual self-employment choice [39–41]. That is, the high regional self-employment rate implied a better entrepreneurship environment, a feature that plays an important role in entrepreneurial orientation [41, 42]. As expected, provincial self-employment rate has been positively related to the self-employment behavior in the result of the first stage of IV-LPM. In Table 6 in Appendix, the F statistic in the first stage of the sub-health status model and the chronic disease model also indicated that the instrument variable was strong (F > 10). We respectively reported Anderson canon. Corr. LM statistic and Cragg–Donald Wald F statistics. The former was jointly significant at the 1% level, which passed unidentified test, and the latter were more than the Stock–Yogo weak ID test critical values at the 10% maximal IV size, which also rejected the null hypothesis of weak IV. Therefore, the instrument variable was valid for correcting endogeneity in this study.

In addition, provincial self-employment rate had no direct influence on the health of rural migrants at the individual level according to the calculation mode. Overall, the instrument variable was orthorhombic with the self-employment of rural migrants in urban destinations and was unrelated to the error term in the main regression model. Consequently, we selected the provincial self-employment rate as our instrument variable.

Giandrea, Cahill and Quinn [37] found that poor health status had negatively impact on individual self-employment decision. Given their finding, the OLS estimates in this study were downward bias. After correcting the bias by using IV-LPM regression, our IV estimates indicated that our findings were in accord with Giandrea, Cahill and Quinn [37] reversed causality story.

## Results

### Descriptive statistics

The resulting descriptive statistics are shown in Table 2. Self-employed rural migrants accounted for 34.83% (*n* = 39,937) of total observations. 15.33% (*n* = 17,085) and 7.39% (*n* = 8474) of the rural migrants experienced sub-health status and chronic disease, respectively. Nearly half of the participants were male migrants, and the average age was approximately 35 years old. Most of the rural migrants completed the nine-year compulsory education, and 32.54% of them also achieved educational attainments over senior high school.

**Table 2:** Descriptive statistics of study participants according to employment status. (NMPDMS-2017)

	Full sample N(%)	Self-employed N(%)	Wage-employed N(%)	***P***-value
**Health status**				
Sub-health status				
Sub-healthy, n (%)	17,085(15.33)	6136(15.68)	10,949(15.14)	0.018
Healthy, n (%)	94,339(84.67)	32,992(84.32)	61,347(84.86)	
Chronic disease				
Have Hypertension or Diabetes, n (%)	8474(7.39)	3378(8.46)	5096(6.82)	0.000
Without Hypertension or Diabetes, n (%)	106,201(92.61)	36,559(91.54)	69,642(93.18)	
**Socio-economic characteristics**				
Gender				
Male, n (%)	57,279(49.95)	23,085(57.80)	34,194(45.75)	0.000
Female, n (%)	57,396(50.05)	16,852(42.20)	40,544(54.25)	
Age mean ± SD	35.372 ± 10.884	37.591 ± 9.533	34.187 ± 11.366	0.000
Age^2, mean ± SD	35.372 ± 10.884	37.591 ± 9.533	34.187 ± 11.366	0.000
Education attainment				
Primary school or below, n (%)	22,301(19.45)	8878(22.23)	13,423(17.96)	0.000
Junior high school, n (%)	55,056(48.01)	21,787(54.55)	33,269(44.51)	0.000
Senior high school, n (%)	24,070(20.99)	7376(18.47)	16,694(22.34)	0.000
College or above, n (%)	13,248(11.55)	1896(4.75)	11,352(15.19)	0.000
Marital status				
Married, n (%)	96,687(84.31)	37,303(93.40)	59,384(79.46)	0.000
Unmarried, n (%)	17,988(15.69)	2634(6.60)	15,354(20.54)	
Income (monthly), mean ± SD	3962.79 ± 2858.13	4108.99 ± 3461.81	3850.94 ± 2284.99	0.000
**Work characteristics**				
Industry				
Other, n (%)	8352(9.07)	3433(8.60)	4919(9.42)	0.000
Construction industry, n (%)	27,304(29.64)	5953(14.90)	21,351(40.91)	0.000
Service industry, n (%)	56,478(61.30)	30,551(76.50)	25,927(49.67)	0.000
**Migration traits**				
Migrated with children				
Yes, n (%)	74,762(65.19)	30,448(76.24)	44,314(59.29)	0.000
No, n (%)	39,913(64.81)	9489(23.76)	30,424(40.71)	
Migrated with couple				
Yes, n (%)	84,230(73.45)	34,200(85.63)	50,030(66.94)	0.000
No, n (%)	30,445(26.55)	5737(14.37))	24,708(33.06)	
**N, n (%)**	114,675(100)	39,937(34.83)	74,738(65.17)	

*SD* Standard deviation

The baseline characteristics by employment status revealed that the self-employed migrants suffered more health risks than their wage-employed counterparts (sub-health status: 15.68% vs. 15.14%; chronic disease: 8.46% vs. 6.82%). Male self-employed migrants outnumbered female self-employed migrants (57.80% vs. 42.20%), and the average age of the self-employed migrants was higher than that of wage-employed ones (37.59 vs. 34.19). Moreover, the wage-employed had higher education levels than self-employed counterparts (primary school or below: 17.96% vs. 22.23%; junior high school: 44.51% vs. 54.55%; senior high school: 22.34% vs. 18.47%; college or above: 15.19% vs. 4.75%). Moreover, service industry was the most important industry for self-employed migrants, where 76.50% of them worked in; and the self-employed had higher incomes than the wage-employed (RMB 4108.99 vs. RMB 3850.94). Additionally, self-employed migrants had a higher likelihood of migrating with their family. Among those workers, 76.24% migrated with their children, and 85.63% migrated with their spouse.

### Baseline estimation

Two logistic regressions were applied to explore the effect of self-employment on rural migrants’ health status. Sub-health status and chronic disease were considered distinct dependent variables. The results are shown in Table 3.

**Table 3:** Logit regression models assessing the effect of self-employment on sub-health status and chronic disease

	Sub-health status		Chronic disease	
	β	95% CI	β	95% CI
Employment status				
Self-employed	0.0377*	(− 0.0044–0.0798)	0.3401***	(0.2792–0.4011)
Wage-employed	Reference		Reference	
Gender				
Male	−0.1508***	(− 0.1913–−0.1103)	0.1660***	(0.1071–0.2248)
Female	Reference		Reference	
Age	0.0265***	(0.0124–0.0405)	0.1119***	(0.0916–0.1322)
Age^2	0.0002**	(0.00005–0.0004)	− 0.0004***	(− 0.0007–−0.0002)
Education attainment				
Primary school or below	Reference		Reference	
Junior high school	−0.1764***	(− 0.2271–−0.1257)	− 0.3176***	(− 0.3842–−0.2510)
Senior high school	− 0.1826***	(− 0.2471–−0.1181)	− 0.4113***	(− 0.5038–−0.3188)
College or above	−0.1708***	(− 0.2545–−0.0872)	− 0.7574***	(− 0.9141–−0.6007)
Marital status				
Married	0.2514***	(0.1570–0.3458)	− 0.3578***	(− 0.5029–−0.2128)
Unmarried	Reference		Reference	
Income (monthly)	−0.00004***	(−0.00005–−0.00003)	−0.00003***	(−0.00004–−0.00001)
Industry				
Other	Reference		Reference	
Construction industry	−0.0690*	(− 0.1404–0.0024)	0.0666	(− 0.0361–0.1693)
Service industry	− 0.0616*	(− 0.1285–0.0053)	0.0401	(− 0.0537–0.1339)
Migrated with children				
Yes	0.0101	(− 0.0449–0.0651)	− 0.1220***	(− 0.1955–−0.0484)
No	Reference		Reference	
Migrated with couple				
Yes	−0.0961***	(−0.1653–−0.0269)	− 0.0307	(− 0.1327–0.0713)
No	Reference		Reference	
Constant	−2.8197***	(−3.0807–−2.5588)	−5.8291***	(−6.2506–−5.4076)

*β* Logit regression Coefficient, *CI* Confidence interval

***significant at 1%, **significant at 5%, *significant at 10%

In the sub-health status model, the effect of the key variable was significantly positive, which indicated that the self-employment had a negative impact on the sub-health status of rural migrants (β = 0.0377; 95% CI: − 0.0044, 0.0798). That is, self-employed migrants were more likely to suffer from sub-health status than their employed counterparts in China. Meanwhile, being married (β = 0.2514, 95% CI: 0.1570, 0.3458) increased the likelihood of suffering from sub-health status. By contrast, gender  (β = − 0.1508, 95% CI: − 0.1913, − 0.1103), educational attainment (junior high school: β = − 0.1764, 95% CI: − 0.2271, − 0.1257; senior high school: β = − 0.1826, 95% CI: − 0.2471, − 0.1181; college or above: β = − 0.1708, 95% CI: − 0.2545, − 0.0872) and income (β = − 0.00004, 95% CI: − 0.00005, − 0.00003) had a negative impact on the sub-health status of rural migrants. Industry factor had a similar result, such that rural migrants working in construction and service industries were less likely to suffer from sub-health status than their counterparts working in other industries (construction industry: β = − 0.0690, 95% CI: − 0.1404, 0.0024; service industry: β = − 0.0616, 95% CI: − 0.1285, 0.0053). In the migration trait, migrating couples (β = − 0.0961, 95% CI: − 0.1653, − 0.0269) was less likely to experience sub-health status.

The chronic disease model established that self-employment increased the likelihood of suffering from chronic disease for rural migrants (β = 0.3401, 95% CI: 0.2792, 0.4011). Being male (β = 0.1660, 95% CI: 0.1071, 0.2248), unmarried (β = − 0.3578; 95% CI: − 0.5029, − 0.2128), low income (β = − 0.00003, 95% CI: − 0.00004, − 0.00001), and older rural migrants (β = 0.1119, 95% CI: 0.0916, 0.1322) were more likely to suffer from chronic disease. By contrast, rural migrants who migrated with their children (β = − 0.1220, 95% CI: − 0.1955, − 0.0484) were less likely to suffer chronic disease.

### IV-LPM estimation

IV-LPM was applied to correct the endogeneity of self-employment. Table 4 showed LPM and IV-LPM estimations. The results indicated that self-employment still had a significantly negative effect on rural migrants’ sub-health status and chronic disease even when we corrected the potential endogeneity by using IV-LPM regression. Self-employed migrants increased the likelihood that their sub-health status was bad by 0.47% or 2.4% and chronic disease was bad by 1.99% or 2.77%, depending on the models. After closely examining the estimates from LPM and IV-LPM models, we found that results of the former were smaller in sub-health status model (0.47% compared with 2.4%) and chronic disease model (1.99% compared with 2.77%), which were in accord with the reserve causality story.

**Table 4:** Causal effect between self-employment and health status among rural migrants: LPM and IV-LPM regression models

	Sub-health status		Chronic disease	
	LPM	IV-LPM	LPM	IV-LPM
Employment status				
Self-employed	0.0047* (− 0.0004, 0.0097)	0.0240**(0.0047, 0.0433)	0.0199***(0.0163, 0.0235)	0.0277***(0.0144, 0.0410)
Wage-employed	Reference		Reference	
Gender				
Male	−0.0179*** (− 0.0226, − 0.0132)	−0.0183***(− 0.0230, − 0.0136)	0.0090***(0.0058, 0.0121)	0.0088***(0.0056, 0.0120)
Female	Reference		Reference	
Age	−0.0037*** (− 0.0056, − 0.0019)	−0.0041***(− 0.0058, − 0.0023)	−0.0063***(− 0.0078, − 0.0049)	−0.0065***(− 0.0076, − 0.0053)
Age^2	0.0001*** (0.0001, 0.0001)	0.0001***(0.0001, 0.0001)	0.0001***(0.0001, 0.0002)	0.0001***(0.0001, 0.0001639)
Education attainment				
Primary school or below	Reference		Reference	
Junior high school	−0.0275*** (− 0.0350, − 0.0200)	−0.0266***(− 0.0333, − 0.0200)	−0.0272***(− 0.0330, − 0.0215)	−0.0269***(− 0.0314, − 0.0223)
Senior high school	−0.0277*** (− 0.0361, − 0.0193)	−0.0252***(− 0.0334, − 0.0171)	−0.0300***(− 0.0362, − 0.0238)	−0.0290***(− 0.0346, − 0.0234)
College or above	− 0.0254*** (− 0.0348, − 0.0161)	−0.0198***(− 0.0307, − 0.0090)	−0.0337***(− 0.0401, − 0.0273)	−0.0315***(− 0.0389, − 0.0240)
Marital status				
Married	0.0256*** (0.0161, 0.0352)	0.0241***(0.0138, 0.0344)	−0.0049(− 0.0110, 0.0012)	−0.0055(− 0.0126, 0.0016)
Unmarried	Reference		Reference	
Income (monthly)	−4.09E-06***(−4.88E-06, −3.31E-06)	--4.32E-06***(− 5.16E-06, −3.48E-06)	−1.29E-06***(− 1.89E-06, −6.93E-07)	−1.38E-06***(− 1.96E-06, −8.04E-07)
Industry				
Other	Reference		Reference	
Construction industry	−0.0078* (− 0.0167, 0.0010)	− 0.0051(− 0.0141, 0.0039)	0.0043(− 0.0020, 0.0106)	0.0054*(− 0.0007, 0.0116)
Service industry	−0.0069 (− 0.0154, 0.0016)	−0.0103**(− 0.0191, − 0.0016)	0.0031(− 0.0030, 0.0092)	0.0017(− 0.0042, 0.0077)
Migrated with children				
Yes	0.0013 (− 0.0060, 0.0086)	0.0009(− 0.0059, 0.0078)	− 0.0101***(− 0.0155, − 0.0047)	−0.0102***(− 0.0149, − 0.0056)
No	Reference		Reference	
Migrated with couple				
Yes	−0.0121*** (− 0.0208, − 0.0033)	−0.0141***(− 0.0226, − 0.0056)	−0.0004(− 0.0064, 0.0055)	−0.0012(− 0.0071, 0.0046)
No	Reference		Reference	
Constant	0.1478*** (0.1165, 0.1790)	0.1501***(0.1200, 0.1802)	0.1121***(0.0881, 0.1360)	0.1129***(0.0925, 0.1334)

***significant at 1%, **significant at 5%, *significant at 10%

In terms of sub-health status, the IV-LPM estimation revealed that male (β = − 0.0183, 95% CI: − 0.0230, − 0.0136) and unmarried (β = 0.0241, 95% CI: 0.0138, 0.0344) rural migrants were less likely to be in sub-health status, and those who migrated as a couple (β = − 0.0141, 95% CI: − 0.0226, − 0.0056) had less possibility to be in sub-health status. In addition, income (β = − 4.32E-06, 95% CI: − 5.16E-06, − 3.48E-06) and educational attainment (junior high school: β = − 0.0266, 95% CI: − 0.0333, − 0.0200; senior high school: β = − 0.0252, 95% CI: − 0.0334, − 0.0171; college or above: β = − 0.0198, 95% CI: − 0.0307, − 0.0090) had a negative impact on the likelihood of suffering sub-health status.

In the chronic disease model, male migrants (β = 0.0088, 95% CI: 0.0056, 0.0120) were more susceptible to chronic disease, but migrants with higher educational attainment (junior high school: β = − 0.0269, 95% CI: − 0.0314, − 0.0223; senior high school: β = − 0.0290, 95% CI: − 0.0346, − 0.0234; college or above: β = − 0.0315, 95% CI: − 0.0389, − 0.0240) and income (β = − 1.38E-06, 95% CI: − 1.96E-06, − 8.04E-07) had a lower likelihood of suffering from chronic disease. Furthermore, rural migrants who migrated with their children (β = − 0.0102, 95% CI: − 0.0149, − 0.0056) were less likely to suffer from chronic disease.

### Mechanism analysis

Why does self-employment show a negative impact on the health status of rural migrants? This study claimed that social health insurance may serve as the potential mechanism linking self-employment behavior and rural migrants’ health in China. That is, self-employment influenced rural migrants’ health via the access to health services determined by the enrollment in social health insurance. To investigate the transmission channel, we explored the relationship between self-employment and social health insurance. Table 5 revealed that the self-employed in urban destinations was less likely to participate in social health insurance (β = − 2.6891, 95% CI: − 2.7559, − 2.6223).

**Table 5:** Logit regression models assessing the relationship between self-employment and social health insurance

	Social health insurance	
	β	95% CI
Employment status		
Self-employed	−2.6891***	(−2.7559, − 2.6223)
Wage-employed	Reference	
Gender		
Male	−0.0505**	(−0.0906, − 0.0104)
Female	Reference	
Age	0.1847***	(0.1682, 0.2012)
Age^2	−0.0024***	(−0.0026, − 0.0021)
Education attainment		
Primary school or below	Reference	
Junior high school	0.7024***	(0.6303, 0.7744)
Senior high school	1.3755***	(1.2976, 1.4534)
College or above	2.3361***	(2.2518, 2.4204)
Marital status		
Married	0.4233***	(0.3482, 0.4984)
Unmarried	Reference	
Income (monthly)	0.0001***	(0.00008, 0.00009)
Industry		
Other	Reference	
Construction industry	0.8963***	(0.8241, 0.9685)
Service industry	0.1161***	(0.0436, 0.1886)
Migrated with children		
Yes	−0.0228	(−0.0807, 0.0350)
No	Reference	
Migrated with couple		
Yes	−0.3237***	(−0.3881, − 0.2593)
No	Reference	
Constant	−5.9795***	(−6.2675, −5.6916)

*β* Logit regression Coefficient, *CI* Confidence interval

***significant at 1%, **significant at 5%, *significant at 10%

## Discussion

The estimations from the logit regression and IV-LPM estimation confirmed that the self-employed were more susceptible to suffer from sub-health status and chronic disease, an outcome that implied that self-employment activities are not conducive to good health. This finding was in line with that of Rietveld, Kippersluis, and Thurik [35] who revealed a negative effect of self-employment on health status. Several reasons explained the negative relationship between the self-employment and health status of rural migrants in China. First, self-employment was a “double-edged sword” [11] that endowed autonomy and independence, but was accompanied by considerable uncertainty and market fluctuations. In general, self-employed migrants in China encountered numerous difficulties in starting a business, such as the lack of access to financial services, the tediousness of gaining official approval from authorities, complex business registration process, and the multitude of tax items involved [43, 44]. Those migrants need to be self-dependent and take on extreme pressure to survive, a condition that would impair their physical and mental health [45]. Additionally, the self-employed usually undertook more excessive work load than their waged counterparts [11], and this circumstance would minimize their leisure time and reduce health-promotion activities [20, 46]. In addition, long working hours would break their work–life balance and cause them to suffer from more tension or anxiety, thereby possibly generating sub-health outcomes [47, 48]. These disadvantages from self-employment might increase the risks of poor health status among rural migrants.

The result of mechanism analysis revealed that the self-employed were less likely to enroll in social health insurance, a situation which may lead to insufficient medical service if they become sick. Such a service would be detrimental to their health recovery. This result may be attributed to the unique public health insurance systems in China. In urban destinations, self-employed rural migrants were only eligible to participate in the project Urban Employee Basic Medical Insurance (UEBMI) [49]. The UEBMI for the wage-employed was jointly financed by employers and employees. By contrast, the self-employed were required to pay the insurance premium for themselves, and the resulting costs accounted for 5–8% of the average monthly wage of local residents. This cost was a heavy burden for the self-employed migrants. Therefore, respondents had to give up the rights to participate in basic social health insurance. Without enrollment in the urban social health insurance system, self-employed rural migrants needed to shoulder the entire cost of health services on their own, a circumstance that forced them turn to informal and insufficient health services, such as unsupervised self-medication, medical advice from unlicensed private clinics, or simply endure minor illnesses without seeking any health services.

Self-employment might be linked to worse health outcomes, whereas the lack of health insurance among self-employed migrants may hamper their access to formal health care, thereby inducing poorer health status and higher depression related to self-employment. This interesting finding diverged from the evidence from the US. A few studies on the relationship between self-employment and health status in the US revealed that self-employment does not impact the health status of the self-employed, even if they lacked health insurance [26, 50]. The potential explanation for this contradiction was that self-employed people in the US can access equal health care services through self-insurance. Consequently, self-employment was merely negatively associated with having diabetes and hypertension, but was not significantly associated with negative mental health outcomes in the US context. In the Chinese counterpart, self-employed migrants remained vulnerable groups [51] and had limited ability to afford self-insurance using their own earning or savings. Thus, self-employed migrants in China were more likely to suffer from sub-health status and chronic disease in the absence of public health insurance.

### Limitations

This study explored the causal effect of self-employment on the sub-health status and chronic disease of rural migrants. Unfortunately, we could not explore the long-term relationship between self-employment and sub-health status as well as chronic diseases for rural migrants because of the lack of longitudinal data. Although this work employed the IV-LPM estimation to correct the endogeneity of self-employment, sub-health status and chronic disease may arise from self-employment in the long-term. Therefore, longitudinal data should be used to explore the effect in future studies. Additionally, due to the limitation of the dataset, we couldn’t explore the effect of self-employment on hypertension and diabetes of rural migrants, respectively, which can be done in future research.

## Conclusion

This study discussed the causal effect of self-employment on rural migrants’ health in the context of China using the dataset from the NMPDMS-2017. After correcting the endogeneity, the results confirmed that the self-employed were more likely to suffer sub-health status and chronic disease, and self-employment behavior exerted a harmful effect on rural migrants’ health. Social health insurance may also serve as the transmission channel linking self-employment and rural migrants’ health. That is, the self-employed were less prone to participate in urban health insurance programs, thereby inducing insufficient health services for maintaining health.

This conclusion offered implications. The government should play an important role in enhancing the entrepreneurial climate to enlarge the financial access and remove institutional barriers to the self-employment of rural migrants. Public health service should be provided equally for self-employed rural migrants, including expanding the coverage of urban social health insurance programs and improving the reimbursement levels.

## Data Availability

The NMPDMS was open and available dataset from the Migrant Population Service Center of National Health Commission, the People’s Republic of China (PRC). NMPDMS was however available from the Migrant Population Service Center of National Health Commission upon reasonable research request. NMPDMS could be requested from the website of Migrant Population Service Center: http://www.chinaldrk.org.cn/wjw/.

## Appendix

**Table 6:** Estimation of province self-employment rate and self-employment: First stage

	Sub-health status		Chronic disease	
	Self-employment		Self-employment	
	β	95% CI	β	95% CI
Self-employment rate	1.0938***	(1.067, 1.120)	1.0932***	(1.067, 1.120)
Gender				
Male	0.0157***	(0.010, 0.022)	0.0150***	(0.009, 0.021)
Female	Reference		Reference	
Age	0.0161***	(0.014, 0.018)	0.0156***	(0.014, 0.018)
Age^2	−0.0002***	(−0.0002, − 0.0001)	−0.0002***	(− 0.0002, − 0.0001)
Education attainment				
Primary school or below	Reference		Reference	
Junior high school	−0.0368***	(−0.045, − 0.028)	−0.0368***	(− 0.045, − 0.029)
Senior high school	−0.1257***	(− 0.136, − 0.116)	−0.1251***	(− 0.135, − 0.115)
College or above	−0.2747***	(− 0.286, − 0.263)	−0.2748***	(− 0.286, − 0.263)
Marital status				
Married	0.0958***	(0.083, 0.109)	0.0964***	(0.087, 0.109)
Unmarried	Reference		Reference	
Income (monthly)	0.00002***	(0.00001, 0.00002)	0.00002***	(0.00001, 0.00002)
Industry				
Other	Reference		Reference	
Construction industry	−0.1084***	(−0.119, −0.098)	−0.1124***	(− 0.123, − 0.102)
Service industry	0.1647***	(0.155, 0.175)	0.1620***	(0.152, 0.172)
Migrated with children				
Yes	0.0094**	(0.001, 0.018)	0.0093**	(0.0008, 0.018)
No	Reference		Reference	
Migrated with couple				
Yes	0.0904***	(0.080, 0.101)	0.0898***	(0.079, 0.100)
No	Reference		Reference	
Constant	−0.4406***	(−0.479, −0.402)	−0.4300***	(−0.468, − 0.392)
Under identification test (Anderson canon. corr. LM statistic)	*P*-value = 0.000		*P*-value = 0.000	
Weak identification test (Crag –Donald Wald F statistic)	F statistic = 6477		F statistic = 6548.51	

*Note: *1.* β* Logit regression Coefficient, *CI* Confidence interval. 2.***significant at 1%, **significant at 5%, *significant at 10%

## Abbreviations

NMPDMS-2017: The National Migrants Population Dynamic Monitoring Survey 2017
IV-LMP: Linear probability model with instrument variable

## Footnotes

1 Additionally, in order to compare the results of IV-LPM, we also estimated linear probability model (LPM) in Table 4.
